# Investigation of the Binding Affinity of a Broad Array of l-Fucosides with Six Fucose-Specific Lectins of Bacterial and Fungal Origin

**DOI:** 10.3390/molecules24122262

**Published:** 2019-06-18

**Authors:** Son Thai Le, Lenka Malinovska, Michaela Vašková, Erika Mező, Viktor Kelemen, Anikó Borbás, Petr Hodek, Michaela Wimmerová, Magdolna Csávás

**Affiliations:** 1 Department of Pharmaceutical Chemistry, University of Debrecen, Egyetem tér 1, H-4032 Debrecen, Hungary; le.thai.son@pharm.unideb.hu (S.T.L.); mezo.erika@science.unideb.hu (E.M.); kelemen.viktor@pharm.unideb.hu (V.K.); borbas.aniko@pharm.unideb.hu (A.B.); 2 Central European Institute of Technology, Masaryk University, Kamenice 5, 625 00 Brno, Czech Republic; malinovska@mail.muni.cz; 3 National Centre for Biomolecular Research, Faculty of Science, Masaryk University, Kotlářská 2, 611 37 Brno, Czech Republic; 4 Department of Biochemistry, Faculty of Science, Charles University, Albertov 2030, 128 40 Prague 2, Czech Republic; michael.vaskova@gmail.com (M.V.); petr.hodek@natur.cuni.cz (P.H.); 5 Department of Biochemistry, Faculty of Science, Masaryk University, Kotlářská 2, 611 37 Brno, Czech Republic

**Keywords:** l-fucosides, multivalency, lectins, glycoclusters, hemagglutination, cystic fibrosis

## Abstract

Series of multivalent α-l-fucoside containing glycoclusters and variously decorated l-fucosides were synthesized to find potential inhibitors of fucose-specific lectins and study the structure-binding affinity relationships. Tri- and tetravalent fucoclusters were built using copper-mediated azide-alkyne click chemistry. Series of fucoside monomers and dimers were synthesized using various methods, namely glycosylation, an azide-alkyne click reaction, photoinduced thiol-en addition, and sulfation. The interactions between compounds with six fucolectins of bacterial or fungal origin were tested using a hemagglutination inhibition assay. As a result, a tetravalent, α-l-fucose presenting glycocluster showed to be a ligand that was orders of magnitude better than a simple monosaccharide for tested lectins in most cases, which can nominate it as a universal ligand for studied lectins. This compound was also able to inhibit the adhesion of *Pseudomonas aeruginosa* cells to human epithelial bronchial cells. A trivalent fucocluster with a protected amine functional group also seems to be a promising candidate for designing glycoconjugates and chimeras.

## 1. Introduction

**Figure 1:**
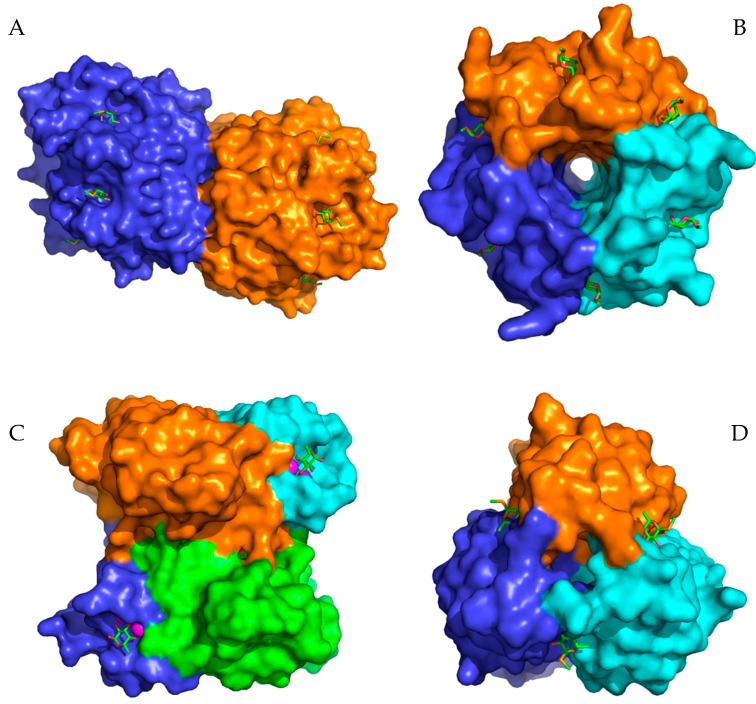
Structures of selected lectins from pathogenic microorganisms used for the inhibition studies. (**A**) AFL dimer in a complex with seleno fucopyranoside (PDB 4AGI). (**B**) RSL trimer in a complex with methyl α-l-fucopyranoside (PDB 2BT9). (**C**) PA-IIL tetramer in a complex with α-l-fucopyranoside (PDB 1UZV). (**D**) Trimer of the BC2L-C N-terminal domain in a complex with seleno fucopyranoside (PDB 2WQ4). Carbohydrates in the binding sites are depicted as sticks. The magenta spheres represent calcium ions in the binding sites of lectin PA-IIL.

Lectins are specific carbohydrate-binding proteins of a non-immune origin. A common role of these proteins is their involvement in recognition and adhesion processes between pathogens and hosts. For example, the chronic infection and colonization of lungs by opportunistic microorganisms is the main cause of mortality among people suffering from cystic fibrosis (CF) [1]. It was demonstrated that major changes in CF glycosylation are represented by increased fucosylation and decreased sialylation [2]. Therefore, lectins from the pathogens could be important virulence factors also representing a suitable therapeutic target [3]. Three of the most widespread pathogens associated with cystic fibrosis—*Pseudomonas aeruginosa*, *Burkholderia cenocepacia*, and *Aspergillus fumigatus*—produce fucose-specific lectins considered to be involved in pathogenesis. The PA-IIL lectin (LecB) from the Gram-negative bacterium *P. aeruginosa* is involved in the adhesion of the bacterium to the host cells via interactions with host glycoconjugates and in the formation of biofilm [4]. PA-IIL was also shown to block epithelial cells’ ciliary beating [5]. The BC2L-C lectin from the bacterium *B. cenocepacia* (closely related to *P. aeruginosa*) has two distinct domains with unique selectivity [6]. The N-terminal domain is a TNF-α-like l-fucose binding domain, while the C-terminal part displays selectivity for d-mannose and l-glycero-d-manno-heptose in a calcium-dependent manner. The N-terminal domain has a strong pro-inflammatory effect and is expected to bind fucosylated epitopes on human glycolipids [6]. *Aspergillus fumigatus* lectin AFL from the fungus *A. fumigatus* stimulates human bronchial cells to produce IL-8 and is supposed to contribute to the inflammatory response observed upon the exposure of a patient to *A. fumigatus* [7]. Although these lectins are all fucose-specific and their supposed functions are similar, they differ significantly in terms of their structural arrangement and binding modes (Figure 1). The N-terminal domain of BC2L-C forms a trimer with binding sites located between neighboring monomers. PA-IIL is a homotetramer with a single binding site per monomer. Two calcium ions mediate binding of the sugar in each binding site [8]. AFL is a dimer, where each monomer forms a six-bladed β-propeller with six non-equivalent binding sites all located on the opposite side of the molecule to the N- and C-termini [9]. 

In this study, we focused on potential inhibitors of fucose-specific lectins from pathogens associated with cystic fibrosis mentioned above. To evaluate common rules for their inhibition, we further included three AFL homologues, known as AAL (*Aleuria aurantia* lectin), AOL (*Aspergillus oryzae* lectin), and RSL (*Ralstonia solanacearum* lectin), from the AAL lectin family, sharing the same six-bladed β-propeller fold, but differing in subtle carbohydrate specificities. The involvement of AOL from *Aspergillus oryzae* in the allergic responses to the fungus was proposed with the suggested mechanism of AOL binding to fucose residues of IgE [10]. AAL from the orange peel fungus *Aleuria aurantia* was the first characterized representative of this lectin family. Both AOL and AAL form dimers similar to AFL. In contrast to the AFL dimer interface, which is formed by loops of all six blades and only the N-terminus is involved, only the loops of blades 6, 1, and 2 come into contact upon dimerization in AAL and both the N- and C-termini are crucial [7,11]. AAL contains only five binding sites, with the sixth believed to be inactive [11]. RSL from *Ralstonia solanacearum,* a dangerous phytopathogen of important agricultural plants (e.g., potatoes, tomatoes) [12], may be involved in adhesion of the bacteria, possibly via binding to terminal fucosides of plant xyloglucans [13]. In contrast to the others, RSL forms the six-bladed β-propeller fold by trimerization, where the monomers present two binding sites each, one formed by oligomerization and the second in between the blades of the same monomer (Figure 1) [13]. 

Lectins are usually multivalent proteins frequently displaying an avidity effect resulting in a significantly increased affinity towards their ligands. Consequently, the multivalent inhibitors with several carbohydrate moieties attached are generally considered to be among the most efficient molecules [14]. Several classes of inhibitors were tested against some of the selected lectins. The known multivalent inhibitors of AFL include cyclopeptide-based hexavalent compounds with a terminal fucose residue and multivalent compounds based on cyclodextrin or octameric silsesquioxane scaffolds [15,16]. C-hexopyranosyl calix[4]arene conjugates were used as potential multivalent inhibitors of BC2L-C and AFL [17]. A broad variety of potential monovalent and multivalent inhibitors were designed and tested against PA-IIL, including C-glycosidic glycomimetics, cinnamide and sulfonamide carbohydrate derivatives, fucofullerenes, glycopeptide dendrimers, pentavalent pillar[5]arene-based glycoclusters, perylenediimide-based glycoclusters, and photoswitchable Janus glycodendrimer micelles [18,19,20,21,22,23,24]. Fucofullerenes and C-hexopyranosyl calix[4]arene conjugates were examined as potential multivalent inhibitors of RSL [17,18,19,20].

In our current work, we focused on multivalent (tri- and tetravalent) glycoclusters with different aglycons (spacers) to exploit their avidity effects. Several thio-α/β-l-fucopyranosides were synthesized to explore the influence of the presence of the anomeric sulfur on the binding, which are more stable in vivo due to the resistance to the degradative enzymes. We further aimed to find a common inhibitor with a reasonable efficiency for all tested lectins from the pathogens associated with cystic fibrosis. The inhibitory potency of all used inhibitors against selected lectins was determined by a hemagglutination inhibition assay with microscope detection, whose robustness and applicability have been demonstrated previously [25,26].

## 2. Results and Discussion

**Scheme 1:**
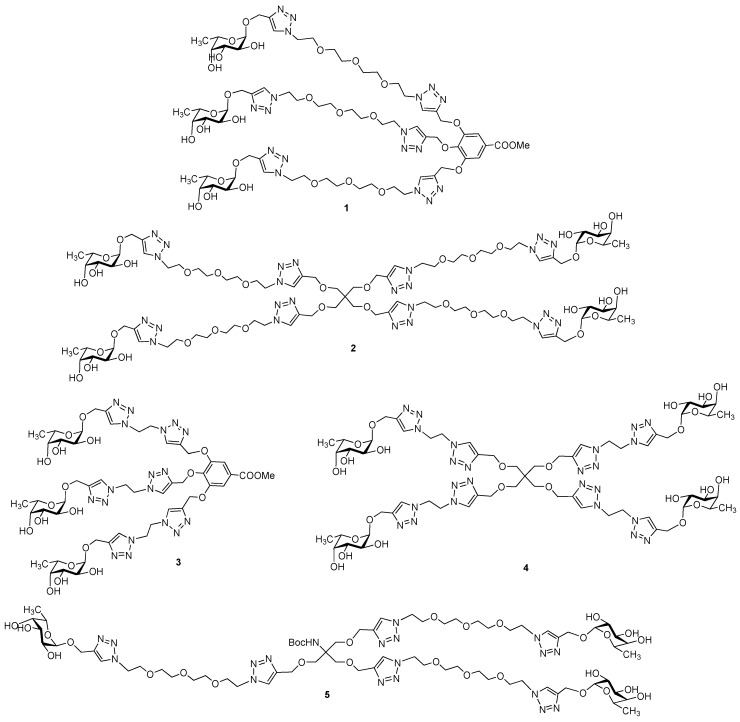
Chemical structures of multivalent glycoclusters containing α-l-fucosides.

**Scheme 2:**
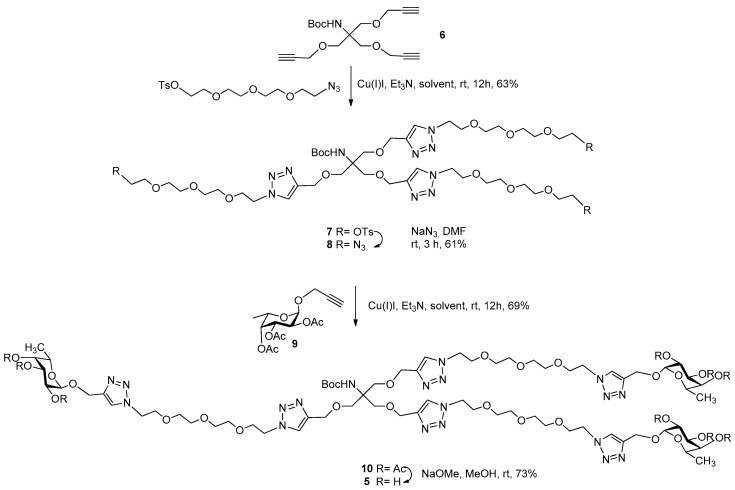
Synthesis of multivalent fucoside **5**.

Tri- and tetravalent α-l-fucose-presenting glycoclusters **1**, **2**, **3**, and **4** were synthesized previously [26] (Scheme 1). Similarly to our recent work [26,27], the fucocluster **5** was synthesized starting from the propargylated NHBoc-Tris scaffold **6** [28] by coupling with tosylated O-(2-azidoethyl)-triethylene glycol [29] using a Cu(I)-catalyzed azide-alkyne cycloaddition reaction (CuAAC) to get tosylated compound **7** (Scheme 2). The tosyl groups of the scaffold were converted into azido functions using sodium azide, resulting in compound **8**, which was also suitable for a subsequent click reaction and was coupled with acetylated propargyl α-l-fucopyranoside **9** [30] to produce the protected fucocluster **10** with a 69% yield. Finally, protecting groups were removed by Zemplén deacetylation to get **5**, containing a protected NH-group, which is suitable for further conjugation to synthesize, e.g., chimera compounds and glycoconjugates.

**Scheme 3:**
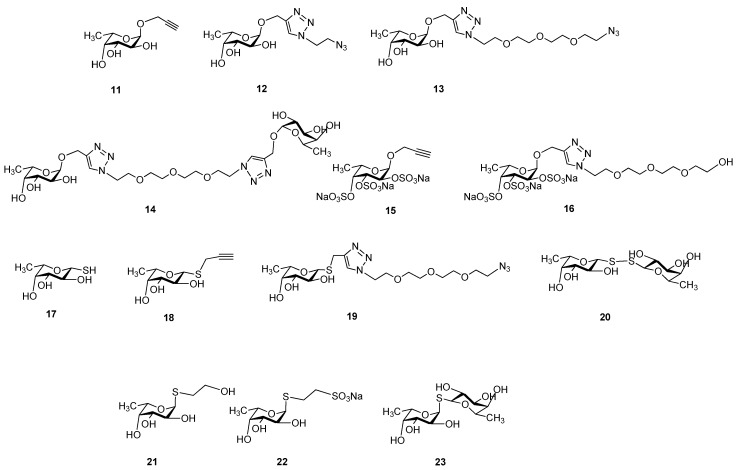
Chemical structures of l-fucoside series.

Other series of already described (**11**, **12**, **13**, **14**, **17**, **18**, and **20**) or newly synthesized fucosides (**15**, **16**, **19**, **21**, **22**, and **23**) were used for an investigation of lectin-carbohydrate interactions that consisted of α-*O*-fucosides (**11** [31], **12** [26], **13** [26], **14** [26]); sulfated α-*O*-fucosides (**15** and **16**); β-1-thiofucose derivatives **17** [32], **18** [33], and **19** and disulfide **20** [34]; as well as α-1-thiofucosides **21**, **22**, and **23** (Scheme 3).

**Scheme 4:**
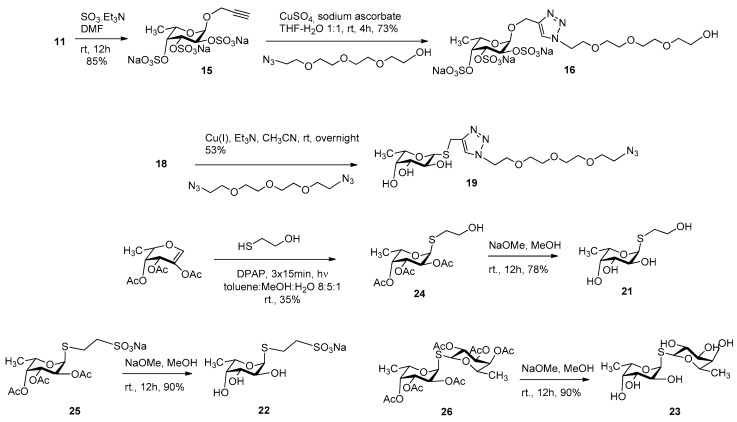
Synthesis of persulfated fucosides **15** and **16** and thioglycosides **19**, **21**, **22**, and **23**.

Persulfation of **11** using the SO_3_.Et_3_N complex resulted in **15** with an 85% yield, which was then coupled with *O*-(2-azidoethyl)triethylene glycol by a click reaction using CuSO_4_-sodium ascorbate to get **16** with a 73% yield (Scheme 4). Compound **19** was prepared from **18** and 1,11-diazido-3,6,9-trioxaundecaneglycol by a Cu(I)-catalyzed click reaction with a 53% yield. Finally, 1,2-cis-α-linked thioglycoside **21** was created in a stereoselective manner by a photoinduced addition reaction [34,35] between 2-acetoxy-3,4-di-*O*-acetyl-l-fucal [36] and 2-mercaptoethanol. The radical-mediated addition provided **24**, which was deacetylated by the Zemplén method, resulting in **21**. *S*-fucoside **22** and thiodisaccharide **23** were prepared by Zemplén deacetylation from their protected forms **25** and **26** [34], respectively.

**Figure 2:**
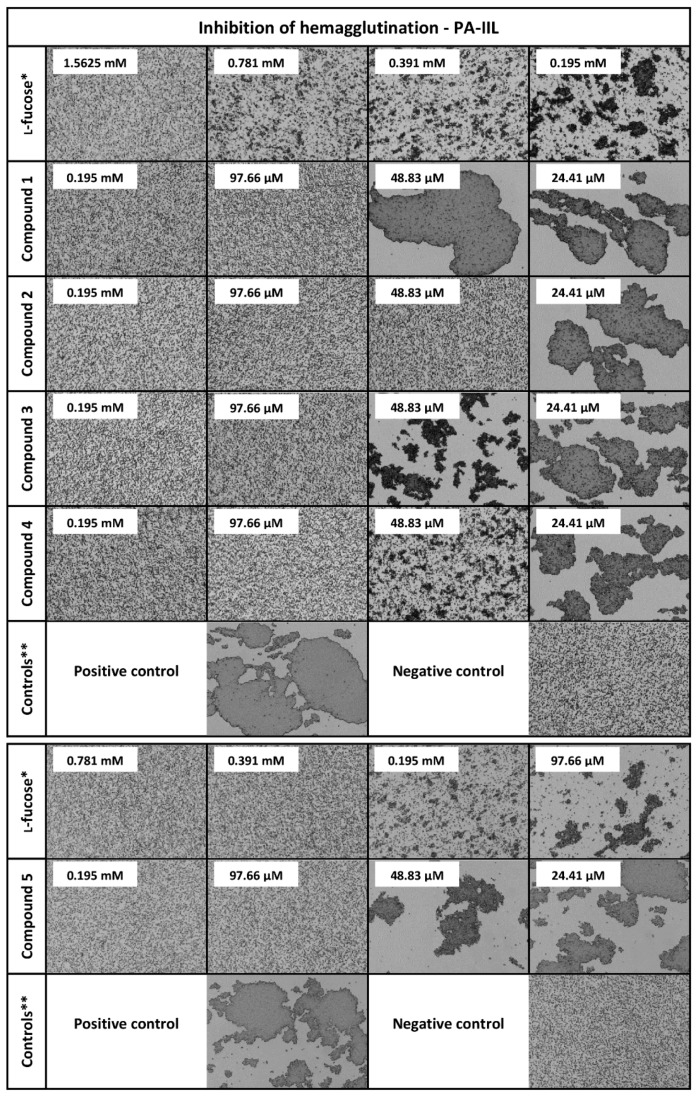
Influence of l-fucose, compounds **1**, **2**, **3**, **4**, and **5**, on hemagglutination caused by lectin PA-IIL. Used as an illustrative example of the data (images) acquired by a hemagglutination inhibition assay with microscope detection. The images for lectins AFL, RSL, AAL, AOL, and BC2L-C are included in the supplementary material. * Standard. Standard experiment was done anew for every used batch of protein or red blood cells. ** Controls were done anew for every used batch of protein or red blood cells. Positive control: experiment without any inhibitor. Negative control: experiment without lectin PA-IIL.

The ability of glycoclusters and l-fucosides to inhibit selected lectins was determined by a hemagglutination inhibition assay with microscope detection (see Table 1 and Figure 2). The inhibitory activity of the tested compounds was semi-quantitatively evaluated by a comparison with the standard (l-fucose). Compounds **15** and **16** were either not able to inhibit any lectin or only very weakly. Although the binding modes of the AAL family, PA-IIL, and BC2L-C differ, the persulfated compounds were not functional inhibitors for any of these proteins, probably due to the steric hindrances and/or requirements of free OH groups for the binding. Compound **17** (thio analogue of l-fucose) was a slightly better inhibitor of PA-IIL, probably due to its small size, but in general, it performed poorly. Lectin BC2L-C was not able to recognize **17** at all. Considering β-thio-l-fucopyranosides, compounds **18** and **19** were generally weak inhibitors with a potency lower than or similar to free l-fucose. The divalent compound **20** was also not an efficient inhibitor of any lectin, confirming the known fact that the simple effect of increasing the number of fucose units is not sufficient for the inhibition [37]. The α-thio-l-fucopyranosides (**21**, **22**, and **23**) were generally better inhibitors than l-fucose for all lectins. It indicated that the weaker binding of **17**, **18**, and **19** was most likely caused by the β configuration and not by the presence of sulfur itself. Therefore, the thioglycosides could be potentially used in the inhibitors to achieve a higher stability towards glycoside hydrolase enzymes. The derivatization of α-l-fucopyranose with different aglycons did not interfere with the inhibitory ability of the tested compounds. The α-*O*-l-fucopyranosides (**11**, **12**, **13**, and **14**) were equal or better inhibitors than l-fucose for all lectins. The used linkers are therefore suitable for usage in multivalent α-l-fucoside-containing glycoclusters. Compounds **12** and **14** were surprisingly very efficient inhibitors of RSL, with a potency of 32 and 128, respectively. This effect was not observed for any other tested lectin, even though AFL, AAL, and AOL are close homologues of RSL. However, RSL differs from other members of the AAL family by displaying a higher affinity to l-fucose. Compound **12** can probably form additional interactions with RSL via its side-chain and the divalent nature of **14** could be enough to exploit the avidity effect of RSL. Considering the multivalent α-l-fucoside-containing glycoclusters, they proved to be efficient inhibitors of lectins from the AAL family. The best inhibitor for all lectins was **2**, the tetravalent compound with longer tetraethylene-glycol spacers. The length of the spacers appeared to be more crucial than the number of branches or their topology. For the majority of the AAL family lectins, the compounds **1**, **2**, and **5** (longer spacers) worked better than compounds **3** and **4** (shorter spacers). The exception to this observation was again for RSL, where **5** represented the least preferred multivalent inhibitor. Considering PA-IIL, compound **2** was also the best inhibitor, even though the binding mode of PA-IIL significantly differs from that of the AAL family. However, the increment of inhibitory potency of multivalent inhibitors compared to the monovalent compounds was lower than for the AAL family. Taking into account the simple effect of the increased concentration of fucose units in the multivalent compound (β, potency/valency), **2** only showed a two times better inhibitory potency than the majority of monovalent compounds (Table 1). **2** was also the best inhibitor of BC2L-C, but its efficiency was even lower. Generally, in the case of BC2L-C, the multivalent compounds used in this study did not display a significantly higher inhibitory potency than monovalent compounds when comparing their β factors. These data may suggest that BC2L-C does not employ a strong avidity effect during interaction. However, as previously tested C-hexopyranosyl calix[4]arene-based inhibitors displayed up to a 256-fold increase in inhibitory potency compared to monovalent l-fucose [17], the currently used multivalent inhibitors may be simply unable to bind to several binding sites simultaneously and further optimization will be necessary. Nevertheless, the tetravalent α-*O*-glycoside **2** with terminal l-fucose with longer spacers surprisingly proved to be the best inhibitor of all lectins used in this study. This universality could be attributed to its flexibility and the sufficient length of the spacers. Although the rigid spacers or scaffolds are generally considered to be more effective for the binding due to the stabilization of optimized sugar conformations and/or due to the lower entropic cost during interaction [37,38], flexible linkers are probably more suitable for the non-specialized inhibition of lectins with different binding modes.

**Figure 3:**
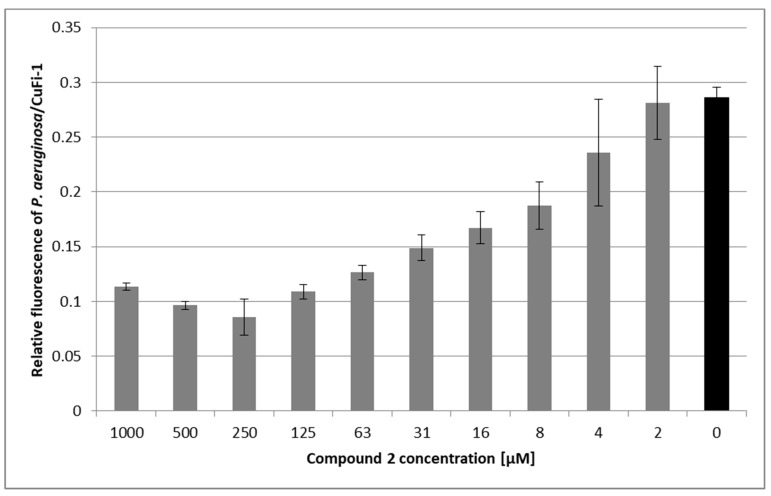
*P. aeruginosa* adhesion to human bronchial epithelial cells (CuFi-1) in the presence of compound **2**. Monolayers of CuFi-1 cells stained with PKH67 were exposed to suspensions containing *P. aeruginosa* stained with PKH26 and compound **2** (grey bars) or PBS (Phosphate Buffered Saline) as a control (black bar). After 2-h incubation, non-adhered bacteria were removed by washing and the relative fluorescence of adhered bacteria and epithelial cells was quantified using Tecan Infinite M200 Pro. Results are expressed as a relative fluorescence ratio of *P. aeruginosa*/CuFi-1 plotted as a function of compound **2**’s concentration. Plotted data are means ± SD of three independent incubations.

As *Pseudomonas aeruginosa* is the most common pathogen associated with cystic fibrosis and involvement of its lectin PA-IIL (LecB) in pathogenesis is continually studied [5,39,40], the inhibitory potential of compound **2** was further evaluated using an ex vivo bacterial adhesion assay. *P. aeruginosa* strain 1763 isolated from a cystic fibrosis patient was incubated with epithelial bronchial cells derived from a cystic fibrosis patient (CuFi-1) in the presence or absence of compound **2**. Ten concentrations of this inhibitor were tested. Compound **2** had a significant protecting effect against *P. aeruginosa* adhesion (see Figure 3). Compared to an untreated control, the inhibitor was able to reduce bacterial adhesion to ∼30% when used in the most effective concentration (0.25 mM). Paradoxically, the inhibitory effect of compound **2** decreased in concentrations above 0.25 mM. This behaviour was attributed to the cross-linking of bacterial cells by a multivalent inhibitor. The subsequent adhesion of bacterial clusters to the epithelial cells resulted in an increased fluorescence signal. Cross-linking of bacterial cells by multivalent carbohydrate-based compounds has been reported previously [17]. In contrast to the low inhibitory activity against lectin PA-IIL observed in vitro by a hemagglutination inhibition assay, the results obtained on a cell-cell level indicate that compound **2** has the potential to reduce the interactions of *P. aeruginosa* with epithelial cells of humans suffering from cystic fibrosis.

Therefore, it could be used for prophylaxis against the bacterial colonization of bronchial cells, similarly to, e.g., chicken immunoglobulins [41], because once the airway cells are colonized, any aid interfering with bacterium adherence is of limited use. As follows from ex vivo experiments with confluent cell layers and a heavy load of *Pseudomonas aeruginosa* (8.4 × 10^6^ CFU/well) presented in this paper, the protective concentration of compound **2** is rather high. It is disputable whether such a concentration is relevant to in vivo scenarios. However, it should be noted that for cystic fibrosis patients, it is feasible to reach high doses of medication applied directly to lungs (inhalation). On a daily basis, they inhale antibiotics, e.g., 300 mg of tobramycin in 5 mL, two times a day, for 28 days [42]. In another study, patients were treated with the inhalation of 10 mL of 0.1 M fucose/0.1 M galactose two times a day for 21 days [43]. Of course, a closer look at in vivo conditions is necessary. A mouse cystic fibrosis model, which is currently being developed [44], is intended to be used.

## 3. Experiment

### 3.1. General Methods

Optical rotations were measured at room temperature with a Perkin-Elmer 241 automatic polarimeter. TLC analysis was performed on Kieselgel 60 F_254_ (Merck, Kenilworth, NJ, USA) silica gel plates with visualization by immersion in a sulfuric-acid solution (5% in EtOH) followed by heating. Column chromatography was performed on silica gel 60 (Merck 0.063–0.200 mm), and flash column chromatography was performed on silica gel 60 (Merck 0.040–0.063 mm). Gel filtration was performed on Sephadex G-25 resin, using water as the eluent. Organic solutions were dried over MgSO_4_ and concentrated under vacuum. The ^1^H (400 MHz) and ^13^C NMR (100.28 MHz) spectra were recorded with a Bruker DRX-400 spectrometer. Chemical shifts are referenced to Me_4_Si or DSS (0.00 ppm for ^1^H) and to solvent signals (CDCl_3_: 77.00 ppm, CD_3_OD: 49.15 ppm, DMSO-d_6_: 39.51 ppm for ^13^C). MS (MALDI-TOF) analysis was carried out in positive reflectron mode with a BIFLEX III mass spectrometer (Bruker, Germany) with delayed-ion extraction. The matrix solution was a saturated solution of 2,5-dihydroxy-benzoic acid (DHB) in *N*,*N*-dimethylformamide.

### 3.2. Synthesis

Compound **8**

Et_3_N (190 µL, 1 equiv./alkyne) and Cu(I)I (2.5 mg, 0.1 equiv./alkyne) were added to a stirred solution of **6** (466 mg, 1.39 mmol) and 2-(2-(2-(2-azidoethoxy)ethoxy)ethoxy)ethyl 4-methylbenzenesulfonate [29] (2.34 g, 6.25 mmol, 1.3 equiv./alkyne) in CH_3_CN (5 mL) under an argon atmosphere and the mixture was stirred overnight at room temperature. The reaction mixture was evaporated, and the crude product was purified by flash column chromatography (CH_2_Cl_2_:MeOH 97:3) to give **7** (1.24 g, 63%) as a yellowish syrup. Subsequently, NaN_3_ (333 mg, 6 equiv.) was added to a stirred solution of **7** (1.24 g, 0.85 mmol) in DMF (10 mL) under an argon atmosphere and stirred overnight at room temperature. The reaction mixture was diluted with water (5 mL), stirred for a further 5 min, and evaporated. The residue was dissolved in CH_2_Cl_2_ (300 mL) and extracted with water (2 × 50 mL) and brine (50 mL), dried over MgSO_4_, filtered, and evaporated. The crude product was purified by flash column chromatography (CH_2_Cl_2_/MeOH 97:3) to give **8** (557 mg, 61%) as a colorless syrup. R_f_ = 0.52 (CH_2_Cl_2_:MeOH 95:5). ^1^H NMR (400 MHz, CDCl_3_) δ 7.70 (s, 3H, 3 × CH triazole), 4.57 (s, 6H), 4.52 (t, *J* = 5.1 Hz, 6H), 3.87-3.85 (t, *J* = 5.1 Hz, 6H), 3.72 (s, 6H, 3 × CH_2_), 3.65–3.60 (m, 28H, 12 × CH_2_), 3.37–3.35 (m, 4H), 2.88 (d, 2H), 2.30–2.25 (m, 2H) 1.37 (s, 9H, 3 × CH_3_). ^13^C NMR (101 MHz, CDCl_3_) δ 154.7 (1C, CO), 144.6 (3C, C=CH), 123.6 (3C, C=CH), 70.5 (3C, 3 × CH_2_), 70.4, 69.9, 69.3 (18C, 18 × CH_2_ TEG), 69.1, 64.7 (3C, 3 × CH_2_), 58.3, 50.5, 50.0 (6C, 6 × NCH_2_ TEG), 28.2 (3C, 3 × CH_3_ Boc); MALDI-TOF MS: *m*/*z* 1090.46 [M + Na]^+^ (calcd for C_42_H_73_N_19_O_14_ 1090.55).

Compound **10**

Et_3_N (73 µL, 1 equiv./alkyne) and Cu(I)I (10 mg, 0.1 equiv./alkyne) were added to a stirred solution of **9** (770 mg, 2.34 mmol) and azide **8** (557 mg, 0.52 mmol, 1.3 equiv./alkyne) in CH_3_CN (5 mL) under an argon atmosphere and the mixture was stirred overnight at room temperature. The reaction mixture was evaporated, and the crude product was purified by flash column chromatography (CH_2_Cl_2_:MeOH 97:3) to give **10** (734 mg, 69%) as a colorless syrup. [α]^24^_D_ -68.18 (c = 0.22, CHCl_3_), R_f_ = 0.38 (CH_2_Cl_2_:MeOH 95:5). ^1^H NMR (360 MHz, CDCl_3_) δ 7.73 (s, 6H, 6 × CH triazole), 5.37-4.10 (m, 15H, 3 × H-1, H-2, H-3, H-4), 4.82 (d, *J* = 12.4 Hz, 3H) 4.66 (d, *J* = 12.4 Hz, 3H), 4.60-4.53 (m, 15H), 4.25-4.19 (m, 3H) 3.89 (t, *J* = 5.2 Hz, 14H,), 3.75 (s, 7H), 3.60 (d, *J* = 4.0 Hz, 20H, CH_2_), 2.14, 2.04, 1.9 (s, 27H, 9 × CH_3,ac_), 2.10-2.01 (m, 2H), 1.40 (s, 9H, 3 × CH_3,Boc_), 1.1 (d, *J* = 6.4 Hz, 9H, 3 × C-6).^13^C NMR (91 MHz, CDCl_3_) δ 196.7 (1C, CO_Boc_) 170.4, 170.2, 169.8 (9C, 9 × CO_ac_), 144.3 (6C, 6 × C=CH triazol) 123.8, (6C, 6 × C=CH triazol) 95.4 (3C, C-1), 70.9, 67.8, 67.7, 64.5 (12C, C_skeleton_), 70.3 (18C, 18 × OCH_2_), 70.2 (3C, 3 × OCH_2_), 69.2, 64.5, 61.0 (6C, 6 × OCH_2_), 50.1 (6C, 6 × NCH_2_ TEG), 28.2 (3C, 3 × CH_3_,_Boc_), 20.6, 20.5 (9C, 9 × CH_3,ac_), 15.7 (3C, 3 × C-6). MALDI-TOF MS: *m/z* 2075.16 [M + Na]^+^ (calcd for C_87_H_133_N_19_O_38_ 2074.90).

Compound **5**

To the stirred solution of **10** (90 mg, 0.04 mmol) in dry MeOH (1 mL), a catalytic amount of NaOMe was added (pH ~ 9). The reaction mixture was stirred overnight at room temperature. The reaction mixture was neutralized with Amberlite IR-120 H^+^ ion-exchange resin, filtered, and evaporated, and the crude product was then purified by flash column chromatography (CH_3_CN:H_2_O 7:3) to give compound **5** (53 mg, 73%) as a white powder. [α]^24^_D_ −27.3 (c = 0.22, MeOH), R_f_ = 0.37 (CH_3_CN:H_2_O 7:3). ^1^H NMR (360 MHz, D_2_O) δ 8.03 (s, 3H, 3 × CH), 7.98 (s, 3H, 3 × CH), 4.95 (bs, 2H), 4.84–4.73 (m, 8H), 4.73–4.63 (m, 8H, 3 × CH_2_), 4.62–4.74 (m, 18H, 3 × H-2, H-3, H-4, H-5), 3.89 (d, *J* = 6.4 Hz, 15H), 3.82–3.69 (m, 9H), 3.52 (dt, *J* = 24.8, 5.0 Hz, 31H), 1.26 (s, 9H, 3 × CH_3_), 1.07 (d, *J* = 6.5 Hz, 9H, 3 × C-6). ^13^C NMR (91 MHz, D_2_O) δ 197.2 (1C, CO_Boc_), 143.8 (6C, 6 × C_q_ triazol), 125.1 (6C, 6 × CH triazol), 98.5 (3C, C-1), 71.7, 69.5, 67.9, 66.7 (12C, C-skeleton), 69.6 (18C, 18 × CH_2_), 69.4 (3C, 3 × CH_2_), 68.6 (1C, C_q_), 63.4, 60.5 (6C, 6 × CH_2_), 58.3 (1C, C_q_), 49.9 (6C, 6 × NCH_2_), 27.5 (3C, 3 × CH_3,Boc_), 15.2 (3C, 3 × C-6). MALDI-TOF MS: *m/z* 1696.91 [M + Na]^+^ (calcd for C_69_H_115_N_19_O_29_ 1696.80).

Compound **15**

Propargyl α-l-fucopyranoside **11** [31] (50 mg, 0.25 mmol) was dissolved in dry DMF (3 mL) and SO_3_^.^Et_3_N (270 mg, 2 equiv./−OH) was then added under argon atmosphere, and it was stirred overnight. The reaction mixture was neutralized with aqueous solution of NaHCO_3_ (250 mg, 6 equiv.) and it was concentrated. The crude product was suspended in MeOH (5 mL) and filtered to remove salts, and was then purified by gel filtration to give compound **15** (107 mg, 85%) as a colorless syrup. [α]^24^_D_ −94.6 (c = 0.13, H_2_O), R_f_ = 0.56 (CH_2_Cl_2_:MeOH:H_2_O 7:6:1). ^1^H NMR (360 MHz, D_2_O) δ 5.39 (d, *J* = 3.8 Hz, 1H), 4.93 (d, *J* = 3.1 Hz, 1H), 4.60–4.51 (m, 2H), 4.40–4.24 (m, 3H), 2.93 (t, *J* = 2.4 Hz, 1H), 1.28 (d, *J* = 6.5 Hz, 3H).^13^C NMR (91 MHz, D_2_O) δ 100.9, 96.6, 79.9, 73.4, 72.9, 67.6, 56.6, 16.6. MALDI-TOF MS: *m/z* 531.18 [M + Na]^+^ (calcd for C_9_H_11_Na_3_O_14_S_3_ 530.89).

Compound **16**

Persulfated propargyl fucoside **15** (50 mg, 0.10 mmol) and O-(2-azidoethyl)triethylene glycol (26 mg, 0.4 mmol) were dissolved in THF:H_2_O = 1:1 (1 mL) and then CuSO_4_ (4 mg, 0.2 equiv.) and Na-ascorbate (20 mg, 1.0 equiv.) were added under argon atmosphere and it was stirred for 4 h. The reaction mixture was concentrated and the crude product was purified by flash column chromatography (CH_2_Cl_2_:MeOH:H_2_O 7:6:1) and by gel filtration to give compound **16** (54 mg, 73%) as a colorless syrup. [α]^24^_D_ −77.0 (c = 0.10, H_2_O), R_f_ = 0.32 (CH_2_Cl_2_:MeOH:H_2_O 7:6:1). ^1^H NMR (400 MHz, D_2_O) δ 8.16 (d, *J* = 3.2 Hz, 1H), 5.32 (dd, *J* = 3.5, 1.5 Hz, 1H), 4.91 (ddq, *J* = 3.1, 2.4, 0.8 Hz, 1H), 4.87–4.82 (m, 2H), 4.71–4.61 (m, 3H), 4.56–4.49 (m, 1H), 4.19–4.11 (m, 1H), 4.01–3.94 (m, 2H), 3.71 (dd, *J* = 6.2, 3.0 Hz, 2H), 3.69–3.59 (m, 11H), 1.19 (dd, *J* = 6.7, 2.9 Hz, 3H). ^13^C NMR (101 MHz, D_2_O) δ 144.6, 126.6, 97.5, 79.8, 73.4, 73.0, 72.6, 70.6, 70.6, 70.4, 69.7, 67.2, 61.9, 61.3, 51.1, 16.6. MALDI-TOF MS: *m/z* 749.95 [M + Na]^+^ (calcd for C_17_H_28_N_3_Na_3_O_18_S_3_ 750.01).

Compound **19**

To a stirred solution of 1,11-diazido-3,6,9-trioxaundecaneglycol (293 mg, 1.2 mmol) and alkyne **18** (138 mg, 0.4 mmol) dissolved in CH_3_CN (5 mL), Et_3_N (60 µL, 1 equiv.) and Cu(I)I (8 mg, 0.1 equiv.) were added under argon atmosphere and it was stirred overnight. The reaction mixture was concentrated and the crude product was purified by flash column chromatography (CH_2_Cl_2_/MeOH 95:5) and gel filtration to give compound **19** (123 mg, 52%) as a colorless syrup. [α]^24^_D_ +29.1 (c = 0.11, MeOH), R_f_ = 0.23 (CH_2_Cl_2_/MeOH 95:5). ^1^H NMR (400 MHz, Methanol-d_4_) δ 7.77 (s, 1H), 4.50 (t, *J* = 5.0 Hz, 2H), 4.28 (d, *J* = 9.5 Hz, 1H), 4.20 (s, 3H), 3.95 (m, 2H), 3.86 (t, *J* = 5.1 Hz, 2H), 3.65 (dd, *J* = 16.2, 7.2 Hz, 12H), 3.48 (dd, *J* = 9.0, 3.4 Hz, 1H), 3.37 (t, *J* = 5.0 Hz, 1H), 3.33 (s, 2H), 1.28 (d, *J* = 6.4 Hz, 3H).^13^C NMR (101 MHz, Methanol-d_4_) δ 145.0, 123.4, 84.8, 74.6, 71.3, 70.2, 70.2, 70.1, 70.1, 69.7, 69.1, 68.9, 50.3, 50.0, 22.7, 16.0. MALDI-TOF MS: *m/z* 485.26 [M + Na]^+^ (calcd for C_17_H_30_N_6_O_7_S 485.18).

Compound **21**

A catalytic amount of NaOMe (pH ~9) was added to a stirred solution of **24** (122 mg, 0.2 mmol) in dry MeOH (3 mL) and stirred overnight at room temperature. The reaction mixture was neutralized with Amberlite IR-120 H^+^ ion-exchange resin, filtered, and evaporated, and the crude product was then purified by flash column chromatography (CH_2_Cl_2_/MeOH 95:5) and gel filtration to give compound **21** (154 mg, 78%) as a colorless syrup. [α]^24^_D_ −331.9 (c = 0.16, MeOH), R_f_ = 0.23 (CH_2_Cl_2_/MeOH 95:5). ^1^H NMR (400 MHz, Methanol-d4) δ 5.36 (d, *J* = 5.6 Hz, 1H), 4.31 (qd, *J* = 6.7, 1.1 Hz, 1H), 4.06 (dd, *J* = 10.1, 5.6 Hz, 1H), 3.80–3.54 (m, 4H), 2.78 (ddd, *J* = 13.5, 7.3, 6.2 Hz, 1H), 2.65 (ddd, *J* = 13.6, 7.3, 6.5 Hz, 1H), 1.25 (d, *J* = 6.6 Hz, 4H). ^13^C NMR (101 MHz, Methanol-d4) δ 88.05 (C-1), 73.39, 72.29, 69.47, 68.07 (C-2,3,4,5), 62.67 (-CH_2_), 33.70 (-SCH_2_), 16.57 (C-6). MALDI-TOF MS: *m/z* 247.32 [M + Na]^+^ (calcd for C_8_H_16_O_5_S 247.06).

Compound **22**

A catalytic amount of NaOMe (pH ~9) was added to a stirred solution of **25** (109 mg, 0.25 mmol) in dry MeOH (10 mL) and stirred overnight at room temperature. The reaction mixture was neutralized with Amberlite IR-120 H^+^ ion-exchange resin, filtered, and evaporated, and the crude product was then purified by flash column chromatography (CH_3_CN/H_2_O 95:5) to give compound **22** (70 mg, 90%) as white crystals. Mp.: 248–249 °C. [α]^24^_D_ −268.3 (c = 0.06, MeOH), R_f_ = 0.53 (CH_3_CN/H_2_O 9:1). ^1^H NMR (360 MHz, Methanol-d4) δ 5.39 (d, *J* = 5.6 Hz, 1H, H-1), 4.38–4.19 (m, 1H), 4.06 (dd, *J* = 10.1, 5.6 Hz, 1H), 3.75–3.54 (m, 2H), 3.18–3.03 (m, 2H), 3.05–2.79 (m, 2H), 1.25 (d, *J* = 6.5 Hz, 3H, CH_3_). ^13^C NMR (91 MHz, Methanol-d4) δ 88.0 (C-1), 73.4, 72.3, 69.4, 68.1 (C-2,3,4,5), 53.2 (-CH_2_SO_3_Na), 26.2 (-SCH_2_), 16.5 (C-6). MALDI-TOF MS: *m/z* 333.11 [M + Na]^+^ (calcd for C_8_H_15_NaO_7_S_2_ 333.01).

Compound **23**

A catalytic amount of NaOMe (pH ~9) was added to a stirred solution of **26** (210 mg, 0.36 mmol) in dry MeOH (10 mL) and stirred overnight at room temperature. The reaction mixture was neutralized with Amberlite IR-120 H^+^ ion-exchange resin, filtered, and evaporated, and the crude product was then purified by flash column chromatography (CH_2_Cl_2_/MeOH 7:3) to give compound **23** (107 mg, 90%) as a white powder. [α]^24^_D_ −179.3 (c = 0.30, MeOH), R_f_ = 0.15 (CH_2_Cl_2_/MeOH 8:2). ^1^H NMR (360 MHz, Methanol-d4) δ 5.60 (d, *J* = 5.5 Hz, 2H, H-1, H-1′), 4.42 – 4.38 (m, 4H), 4.07 (dd, *J* = 10.0, 5.5 Hz, 2H), 3.72–3.35 (m, 8H), 1.26 (d, *J* = 6.4 Hz, 3H), 1.22 (d, *J* = 6.5 Hz, 3H). ^13^C NMR (91 MHz, Methanol-*d*_4_) δ 88.1, 87.6 (C-1, C-1′), 76.3, 76.1, 73.4, 73.1, 72.4, 71.8, 69.7, 68.9 (C-2,3,4,5,2′,3′,4′,5′), 17.2, 16.6 (C-6,6′). MALDI-TOF MS: *m/z* 349.19 [M + Na]^+^ (calcd for C_12_H_22_NaO_8_S 349.09).

Compound **24**

2-Acetoxy-3,4-di-*O*-acetyl-l-fucal (272 mg, 1.0 mmol) and 2-mercaptoethanol (140 µL, 2 mmol) were dissolved in toluene:methanol:water = 8:5:1 (5 mL) and 2,2-dimethoxy-2-phenylacetophenone (DPAP, 25 mg, 0.10 mmol) was added. The solution was irradiated with UV-light at room temperature for 3 × 15 min. Then, the solution was concentrated and the residue was purified using column chromatography (*n*-hexane: aceton 9:1) to give compound **24** (122 mg, 35%) as a colorless syrup. [α]^24^_D_ −135.9 (c = 0.02, CH_3_Cl), R_f_ = 0.50 (*n*-hexane: aceton 7:3). ^1^H NMR (400 MHz, CDCl_3_) δ 5.74 (d, *J* = 5.5 Hz, 1H), 5.37–5.13 (m, 3H), 4.59–4.49 (m, 1H), 3.80 (dt, *J* = 9.0, 4.6 Hz, 2H), 2.94–2.65 (m, 3H), 2.22–2.15 (m, 4H), 2.11 (d, *J* = 1.0 Hz, 3H), 2.20, 2.11, 2.04 (s, 3 × 3H), 1.20 (d, *J* = 6.5 Hz, 3H).

MALDI-TOF MS: *m/z* 373.54 [M + Na]^+^ (calcd for C_14_H_22_O_8_S 373.09).

### 3.3. Lectins Production and Purification

Lectins BC2L-C, AFL, PA-IIL (LecB), and RSL in recombinant forms were produced and purified as previously described [6,7,8,13]. Briefly, transformed *Escherichia coli* cells bearing a plasmid for the particular lectin were cultured in LB (Luria-Bertani) broth medium containing an appropriate antibiotic at 37 °C. When the culture reached an OD_600_ of ≈ 0.5, cells were induced by isopropyl 1-thio-β-d-galactopyranoside (IPTG) added to a final concentration of 0.5 mM. Cells were incubated at 30 °C for 3 h, harvested by centrifugation, and resuspended in a suitable buffer. Cells were then disintegrated by sonication and the cytosolic fraction containing soluble lectin was separated by centrifugation. Lectins were then purified by affinity chromatography on a d-mannose-agarose column, dialyzedm and further processed according to the previously published procedures [6,7,8,13]. Freeze-dried lectins were stored at −20 °C. Lectins AAL (Vector Laboratories) and AOL (TCI) were purchased in a freeze-dried form.

### 3.4. Hemagglutination Inhibition Assay

Lectins AFL, RSL, AAL, and AOL were dissolved in the PBS buffer to the concentration 0.1 mg·mL^−1^. Lectins were mixed with carbohydrate inhibitors serially diluted in PBS buffer in a 5 µL:5 µL ratio. The final (working) concentration of lectins was therefore 0.05 mg·mL^−1^. A total of 10 µL of 20% papain-treated, acid-stabilized red blood cells 0^+^ in PBS buffer was then added, and the mixture was thoroughly mixed and incubated for 5 min at room temperature. After incubation, the mixture was mixed again, transferred to a microscope slide, and examined. The examination was conducted using the Levenhuk D2L NG Digital Microscope (Levenhuk, Tampa, FL, USA). Images were obtained with a Levenhuk D2L digital camera (Levenhuk, Tampa, FL, USA) using the software ToupView for Windows (Levenhuk, Tampa, FL, USA). The positive (experiment without an inhibitor) and negative (experiment without a lectin) control were prepared and processed in the same way using an appropriate volume of dissolving buffer instead of the omitted components. The minimal inhibitory concentration (MIC) of the inhibitor able to inhibit hemagglutination was determined and compared with the standard (l-fucose), and the potency of the inhibitor was calculated (MIC of the standard/MIC of the inhibitor). MIC of the standard was determined every time a new batch of lectins or red blood cells were used for experiments to diminish the biological variability.

The PA-IIL and BC2L-C lectins were dissolved in the buffer containing calcium ions necessary for their activity (20 mM Tris/HCl, 150 mM NaCl, 5 mM CaCl_2_, pH 7.5) to the concentration 2.5 mg·mL^−1^ and 0.2 mg·mL^−1^, respectively. Lectins were mixed with carbohydrate inhibitors serially diluted in the Tris buffer in a 5 µL:5 µL ratio. The final (working) concentration of the lectins was therefore 1.25 mg·mL^−1^ and 0.1 mg·mL^−1^. A total of 10 µL of 20% papain-treated, acid-stabilized red blood cells 0^+^ in the Tris buffer was then added, and the mixture was thoroughly mixed and incubated for 5 min at room temperature. The examination was conducted and evaluated as mentioned above.

The above-mentioned concentrations of all lectins resulted from optimization, i.e., concentrations enabling the formation of observable hemagglutinates in 5 min at room temperature were used.

### 3.5. Pseudomonas Aeruginosa Adherence Assay

#### 3.5.1. Cell Labelling

Epithelial CuFi-1 cells (an immortalized epithelial cell line derived from cystic fibrosis human lungs, ATCC, Poland) were labelled with PKH67 (Fluorescent Cell Linker Kit, Sigma-Aldrich, Taufkirchen, Germany), a green fluorescent membrane marker, as follows. An aliquot of cell suspension containing 7 × 10^6^ cells was washed with PBS buffer and centrifuged (100× *g* for 5 min). The cell pellet was resuspended in 250 µL of Diluent C (Fluorescent Cell Linker Kit, Sigma-Aldrich, Taufkirchen, Germany). Immediately, an equal volume of 8 µM PKH67 in Diluent C (2 µL of PKH67 in 248 µL of Diluent C) was added to the cell suspension. After 5 min incubation at room temperature with periodic mixing, the staining reaction was stopped by adding an equal volume of 1% Fetal Bovine Serum (Gibco^TM^ Invitrogen, Paisley, UK) in PBS. After 1 min incubation with FBS, the suspension was centrifuged (100× *g* for 10 min). Finally, the cells were washed three times with Bronchial Epithelial Cell Growth Basal Medium (Lonza, Basel, Switzerland), followed by centrifugation (100× *g* for 5 min).

*Pseudomonas aeruginosa* strain 1763 (University Hospital Motol, Prague, Czech Republic) was stained with PKH26 (Fluorescent Cell Linker Kit Sigma-Aldrich, Germany), a red fluorescent membrane marker. Bacteria were grown in a suspension culture in PS medium (peptone/casein digest) in an Erlenmeyer flask for 14 h. The culture was washed with PBS buffer and centrifuged (13,400× *g* for 5 min). The pellet containing 1.5 × 10^9^ bacteria was resuspended in 125 µL of Diluent C (Fluorescent Cell Linker Kit, Sigma-Aldrich, Germany) and an equal volume of 16 µM PKH26 in Diluent C (2 µL of PKH26 in 123 µL of Diluent C) was added to the bacterial suspension. The bacteria/dye suspension was incubated for 30 min at room temperature with periodic mixing. The staining was quenched by adding an equal volume of 1% BSA (Bovine Serum Albumin) (Merck, Germany) in PBS. The suspension was incubated with BSA for 1 min to allow the binding of excess dye and then centrifuged (15,700× *g* for 10 min). Bacteria were washed two times with PBS, followed by centrifugation (13,400× *g* for 7.5 and 5 min).

#### 3.5.2. Inhibition of Pseudomonas aeruginosa Adhesion on Epithelial Cells

Epithelial cells CuFi-1 stained with PKH67 were seeded at 8.4 × 10^4^ cells/well onto 96-well plates (CellBIND^®^ 96-well Flat Clear Bottom Black Polystyrene Microplates, Corning Incorporated, Corning, NY, USA) and incubated for 42–45 h at 37 °C, 5% CO_2_, to form a confluent monolayer and regenerate. Prior to the assay, wells were washed with PBS. Compound **2** was diluted with PBS to desired concentrations (2 µM–1 mM). PKH26-stained bacteria *P. aeruginosa* were added to the diluted inhibitor solutions. Bacterial suspensions were immediately added to the wells (50 µL/well). The input ratio was about 100 bacteria per epithelial cell (8.4 × 10^6^ bacteria/well). A suspension without an inhibitor was used as a control. After 2 h incubation at room temperature, the wells were extensively washed three times with PBS to remove non-adhered bacteria The fluorescence of adhered *P. aeruginosa* cells (Ex 522 nm, Em 569 nm for PKH26) on epithelial cells (Ex 470 nm, Em 505 for PKH67) was quantified using the spectrofluorometer Tecan Infinite M200 Pro (Tecan Group Ltd., Männedorf, Switzerland). Results were expressed as a relative fluorescence ratio of *P. aeruginosa*/CuFi-1. Three independent incubations were performed.

## 4. Conclusions

The binding of a broad number of newly synthesized l-fucosides of varying structures, including α- and β-linked mono- and multivalent *O*- and *S*-fucosides, as well as persulfated fucoside derivatives, were tested against six lectins of bacterial and fungal origin. The tetravalent, α-l-fucoside-containing compound with tetraethylene-glycol bridges (**2**) was shown to be a universal inhibitor of fucose-specific lectins from pathogens associated with cystic fibrosis, i.e., *P. aeruginosa*, *B. cenocepacia*, and *A. fumigatus*. This inhibitor was also able to significantly inhibit the adhesion of *P. aeruginosa* cells to epithelial bronchial cells derived from a cystic fibrosis patient in an ex vivo bacterial adherence assay. This molecule also displayed the best inhibitory potency against the RSL lectin from the plant pathogen *R. solanacearum*, suggesting that it could be used to prevent wilting infections of plants. All multivalent inhibitors worked also well against lectins AAL and AOL.

## Figures and Tables

**Table 1:** Potencies of tested inhibitors against fucose-specific lectins determined by a hemagglutination inhibition assay.

		AFL		RSL		AAL		AOL		PA-IIL		BC2L-C	
Inhibitor	Valency	Potency	β^#^	Potency	β^#^	Potency	β^#^	Potency	β^#^	Potency	β^#^	Potency	β^#^
l-fucose*	1	1	1	1	1	1	1	1	1	1	1	1	1
Compound **1**	3	64	21.3	512	170.7	32	10.7	64	21.3	16	5.3	8	2.7
Compound **2**	4	128	32	1024	256	128	32	256	64	32	8	16	4
Compound **3**	3	16	5.3	512	170.7	16	5.3	8	2.7	16	5.3	4	1.3
Compound **4**	4	16	4	256	64	16	4	8	2	16	4	4	1
Compound **5**	3	32	10.7	128	42.7	64	21.3	128	42.7	4	1.3	8	2.7
Compound **11**	1	4	4	8	8	4	4	4	4	4	4	1	1
Compound **12**	1	8	8	32	32	8	8	8	8	4	4	2	2
Compound **13**	1	4	4	8	8	4	4	4	4	4	4	2	2
Compound **14**	2	4	2	128	64	8	4	4	2	8	4	2	1
Compound **15**	1	0.125	0.125	ND	-	ND	-	0.5	0.5	0.5	0.5	ND	-
Compound **16**	1	0.125	0.125	ND	-	ND	-	ND	-	0.125	0.125	0.25	0.25
Compound **17**	1	0.5	0.5	0.5	0.5	0.5	0.5	0.5	0.5	0.25	0.25	ND	-
Compound **18**	1	0.5	0.5	0.5	0.5	1	1	0.5	0.5	0.03125	0.03125	0.5	0.5
Compound **19**	1	1	1	2	2	2	2	1	1	0.0625	0.0625	0.5	0.5
Compound **20**	2	0.5	0.25	2	1	1	0.5	1	0.5	0.125	0.0625	1	0.5
Compound **21**	1	2	2	4	4	2	2	4	4	4	4	4	4
Compound **22**	1	2	2	4	4	2	2	2	2	4	4	4	4
Compound **23**	2	8	4	8	4	4	2	8	4	4	2	8	4

* Standard # Potency/Valency ND Not detected.

## Supplementary Materials

The following are available online: Figure S1: Influence of l-fucose, compounds **1**, **2**, **3**, **4** and **5** on hemagglutination caused by lectin AFL. Figure S2. Influence of l-fucose, compounds **1**, **2**, **3**, **4**, **14** and **5** on hemagglutination caused by lectin RSL. Figure S3. Influence of l-fucose, compounds **1**, **2**, **3**, **4** and **5** on hemagglutination caused by lectin AAL. Figure S4. Influence of l-fucose, compounds **1**, **2**, **3**, **4** and **5** on hemagglutination caused by lectin AOL. Figure S5. Influence of l-fucose, compounds **1**, **2**, **3**, **4** and **5** on hemagglutination caused by lectin BC2L-C.
