# Supplementary material for: Investigation of the Binding Affinity of a Broad Array of l-Fucosides with Six Fucose-Specific Lectins of Bacterial and Fungal Origin
Source: Molecules. 2019 Jun 18;24(12):2262. doi: 10.3390/molecules24122262 (PMC6631993; doi:10.3390/molecules24122262)
Supplement: Supplementary file 1 [file molecules-24-02262-s001.pdf]

Supplementary Information

Investigation of the Binding Affinity of a Broad Array of L-Fucosides with Six Fucose-Specific Lectins of Bacterial and Fungal Origin

Son Thai Le [1,§], Lenka Malinovska[2,3,§], Michaela Vašková[4], Erika Mező[1], Viktor Kelemen[1], Anikó Borbás[1], Petr Hodek[4], Michaela Wimmerová,[2,3,5*] and Magdolna Csávás[1*]

[1] Department of Pharmaceutical Chemistry, University of Debrecen, Egyetem tér 1, H-4032, Debrecen, Hungary; le.thai.son@pharm.unideb.hu (S. T. L.), mezo.erika@science.unideb.hu (E.M.), kelemen.viktor@pharm.unideb.hu (V.K.), borbas.aniko@pharm.unideb.hu (A.B.), csavas.magdolna@science.unideb.hu (M.Cs.)

[2] Central European Institute of Technology, Masaryk University, Kamenice 5, 625 00 Brno, Czech Republic; malinovska@mail.muni.cz (L.M.), michaela.wimmerova@ceitec.muni.cz (M.W.)

[3] National Centre for Biomolecular Research, Faculty of Science, Masaryk University, Kotlářská 2, 611 37 Brno, Czech Republic

[4] Department of Biochemistry, Faculty of Science, Charles University, Albertov 2030, 128 40 Prague 2, Czech Republic, michael.vaskova@gmail.com (M.V.), petr.hodek@natur.cuni.cz (P.H.)

[5] Department of Biochemistry, Faculty of Science, Masaryk University, Kotlářská 2, 611 37 Brno, Czech Republic

[*]Correspondence: michaw@chemi.muni.cz (M.W.); csavas.magdolna@science.unideb.hu (M.Cs.)

[§]These two authors contributed equally.

Received: date; Accepted: date; Published: date

Abstract: Series of multivalent α-L-fucoside containing glycoclusters and variously decorated L-fucosides were synthesized to find potential inhibitors of fucose-specific lectins and study the structure-binding affinity relationships. Tri- and tetravalent fucoclusters were built up using copper-mediated azide-alkyne click chemistry. Series of fucoside monomers and dimers were synthesized using various methods, namely glycosylation, azide-alkyne click reaction, photoinduced thiol-en addition and sulfation. The interactions of compounds with six fucolectins of bacterial or fungal origin were tested using hemagglutination inhibition assay. As a result, a tetravalent, α-L-fucose presenting glycocluster showed to be orders of magnitude better ligand than a simple monosaccharide for tested lectins in most cases, which can nominate it as a universal ligand for studied lectins. This compound was also able to inhibit adhesion of *Pseudomonas aeruginosa* cells to human epithelial bronchial cells. A trivalent fucocluster with protected amine functional group seems also to be a promising candidate to design glycoconjugates and chimeras.

Keywords: L-fucosides, multivalency, lectins, glycoclusters, hemagglutination, cystic fibrosis.

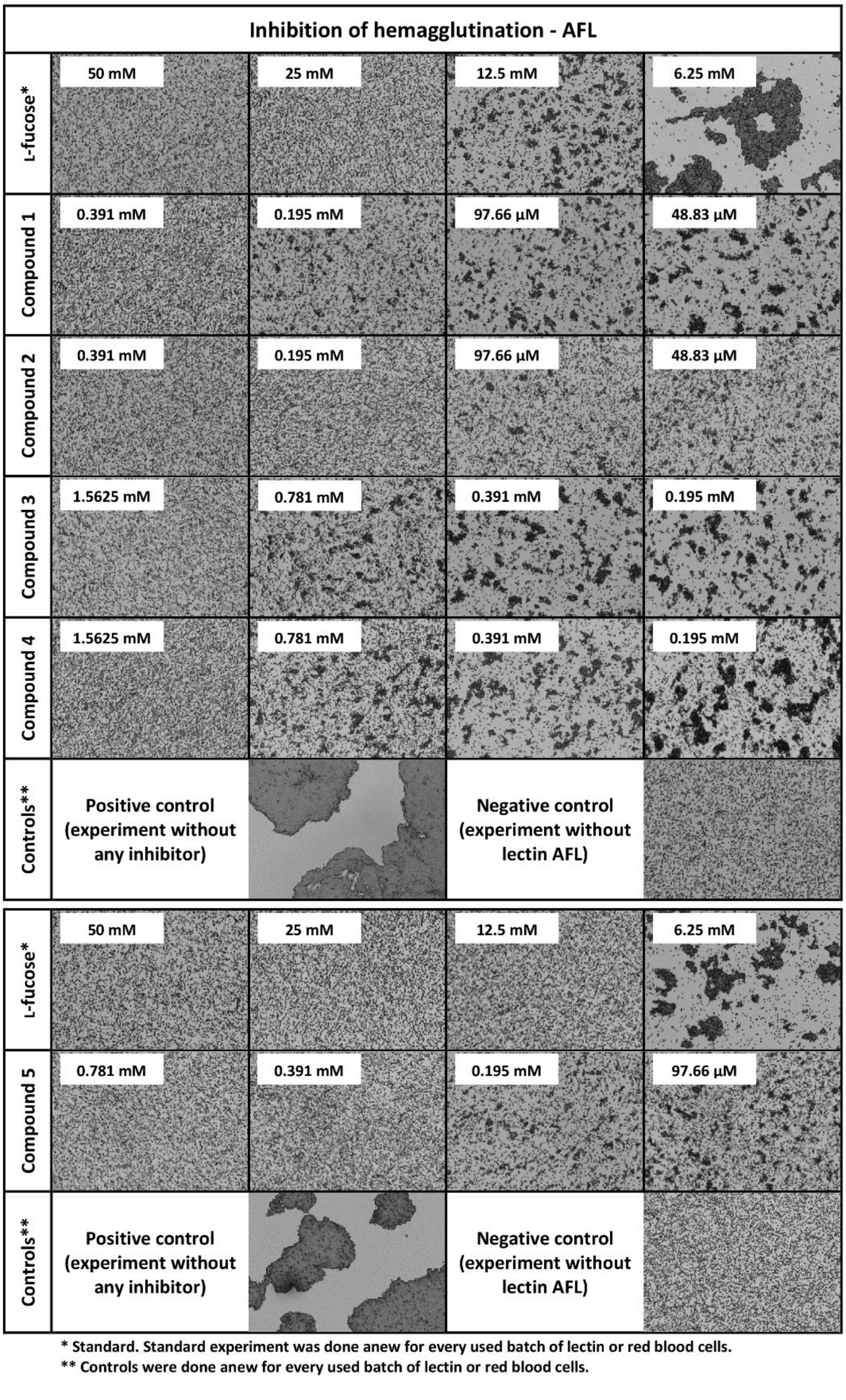

S1. Influence of L-fucose, compounds **1**, **2**, **3, 4** and **5** on hemagglutination caused by lectin AFL.

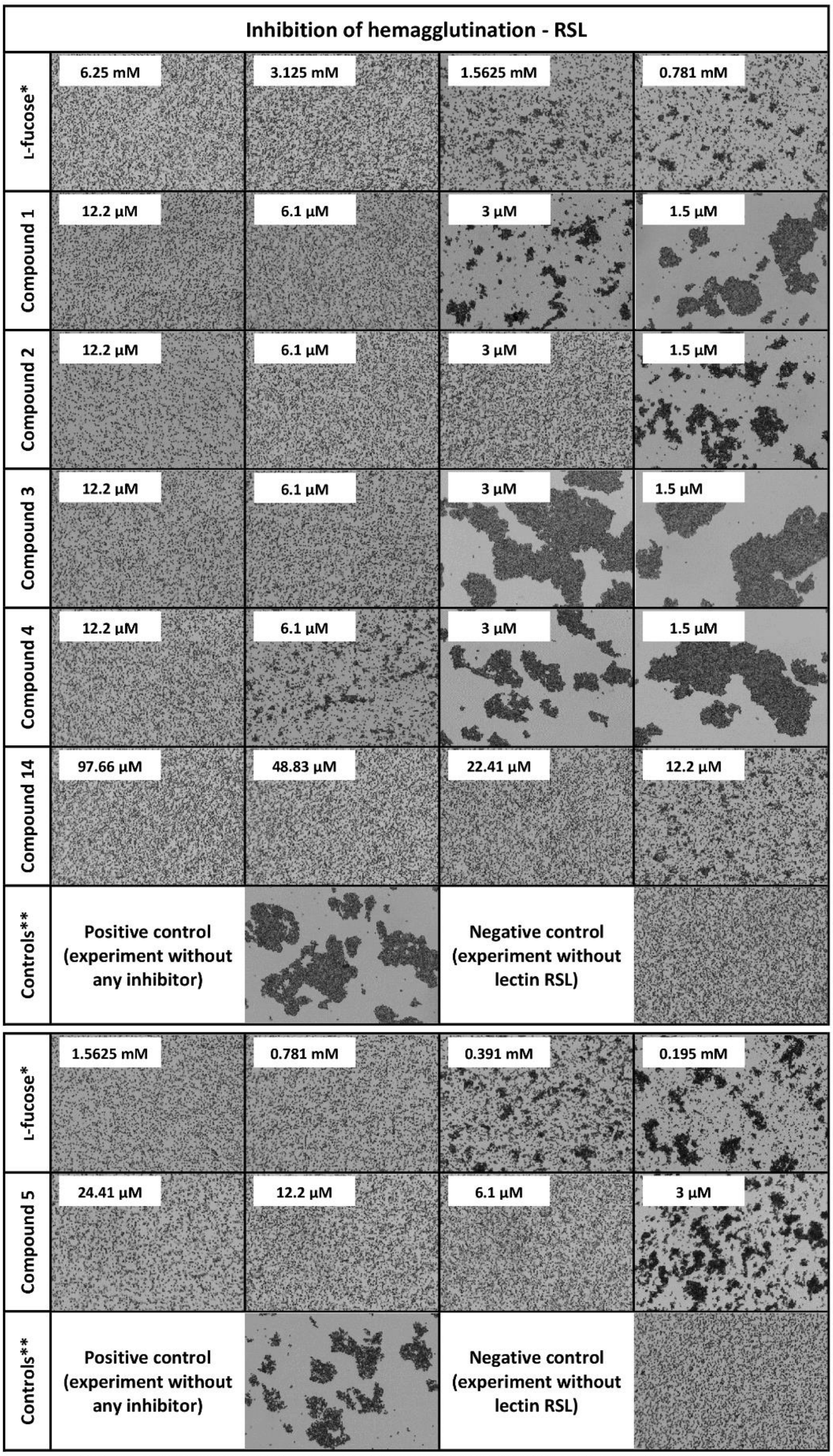

* Standard. Standard experiment was done anew for every used batch of protein or red blood cells.
** Controls were done anew for every used batch of protein or red blood cells.

S2. Influence of L-fucose, compounds **1**, **2**, **3**, **4**, **14** and **5** on hemagglutination caused by lectin RSL.

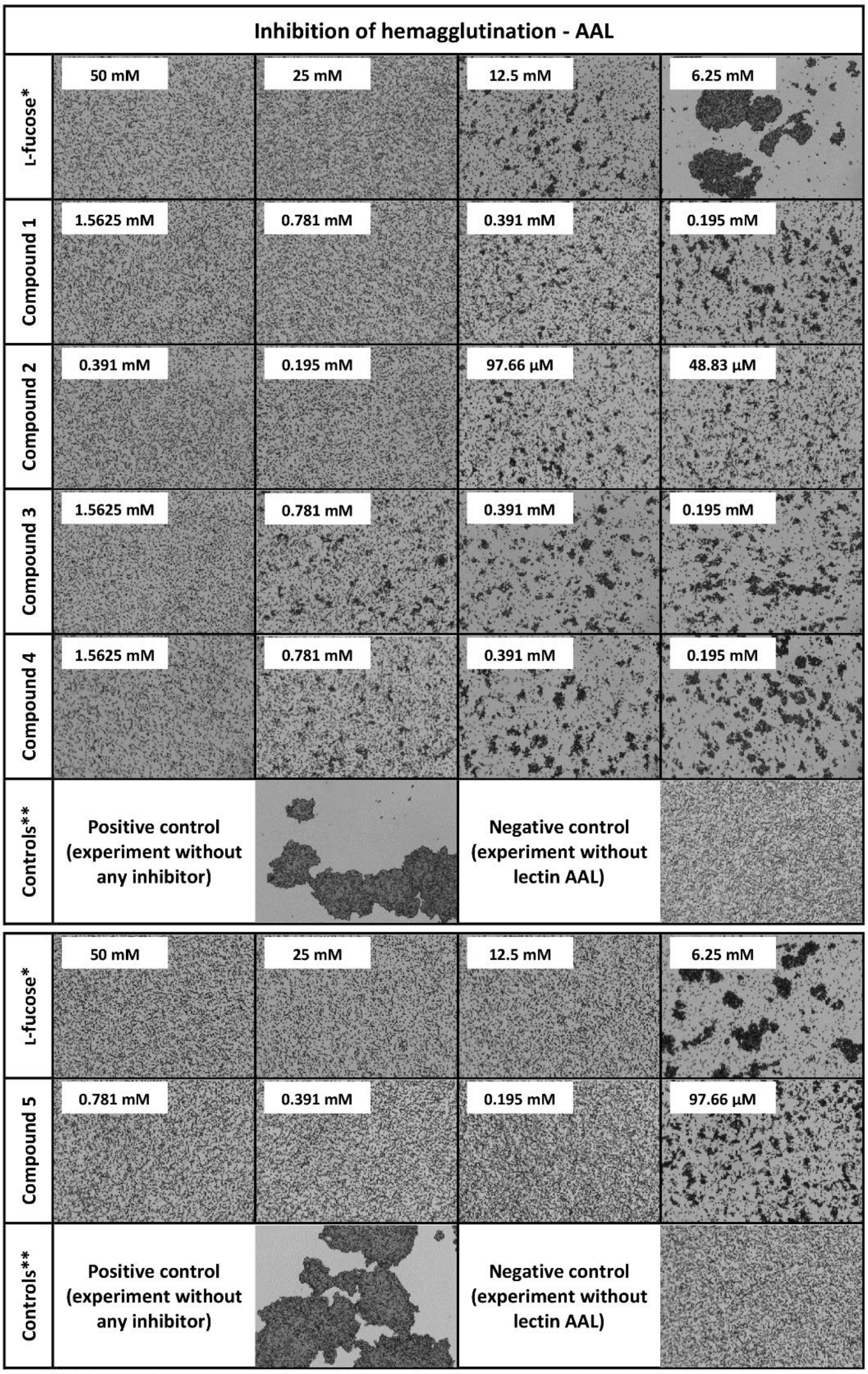

* Standard. Standard experiment was done anew for every used batch of protein or red blood cells.

** Controls were done anew for every used batch of protein or red blood cells.

S3. Influence of L-fucose, compounds **1**, **2**, **3**, **4** and **5** on hemagglutination caused by lectin AAL.

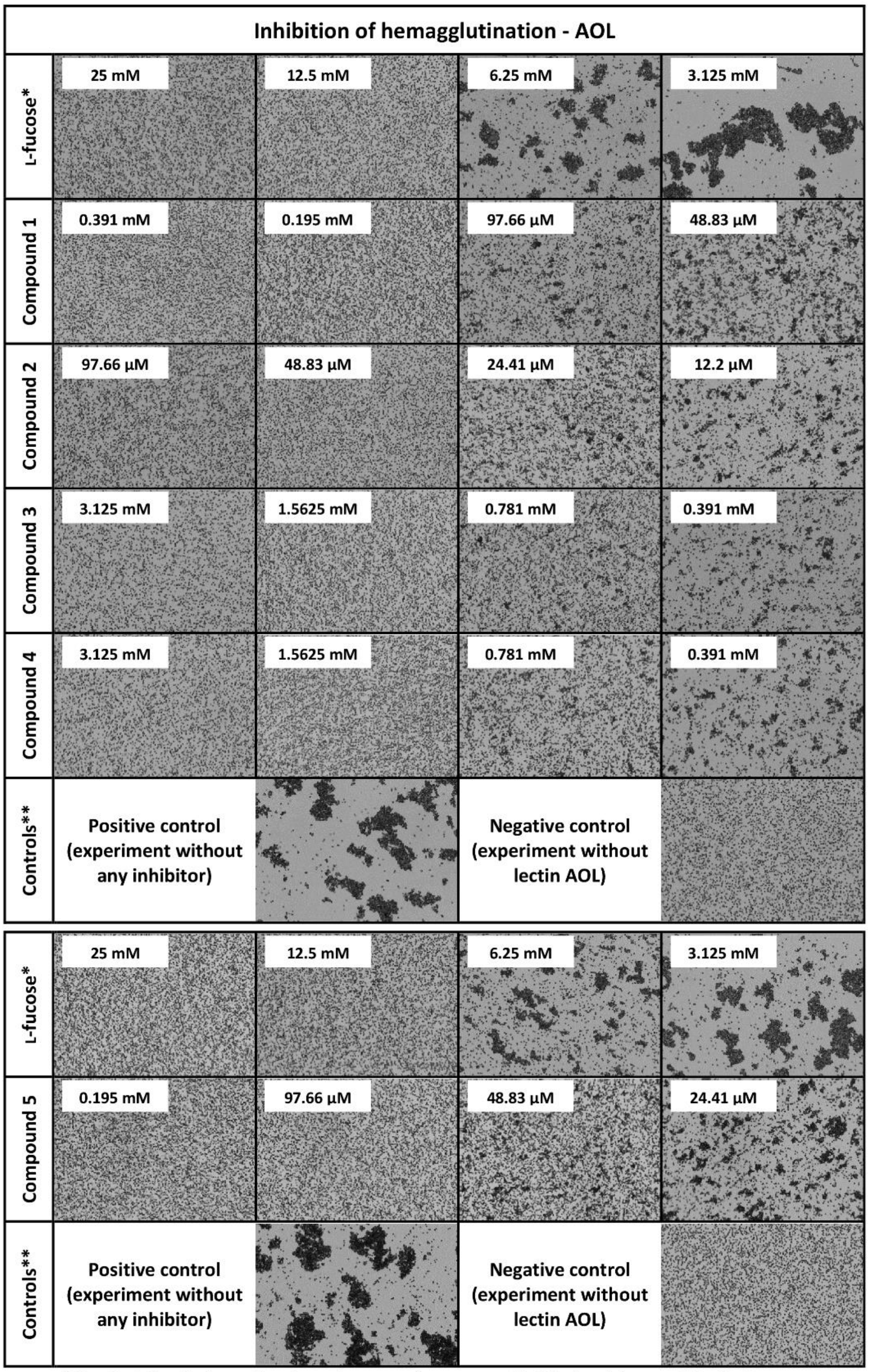

* Standard. Standard experiment was done anew for every used batch of protein or red blood cells.

** Controls were done anew for every used batch of protein or red blood cells.

S4. Influence of L-fucose, compounds **1, 2**, **3**, **4** and **5** on hemagglutination caused by lectin AOL.

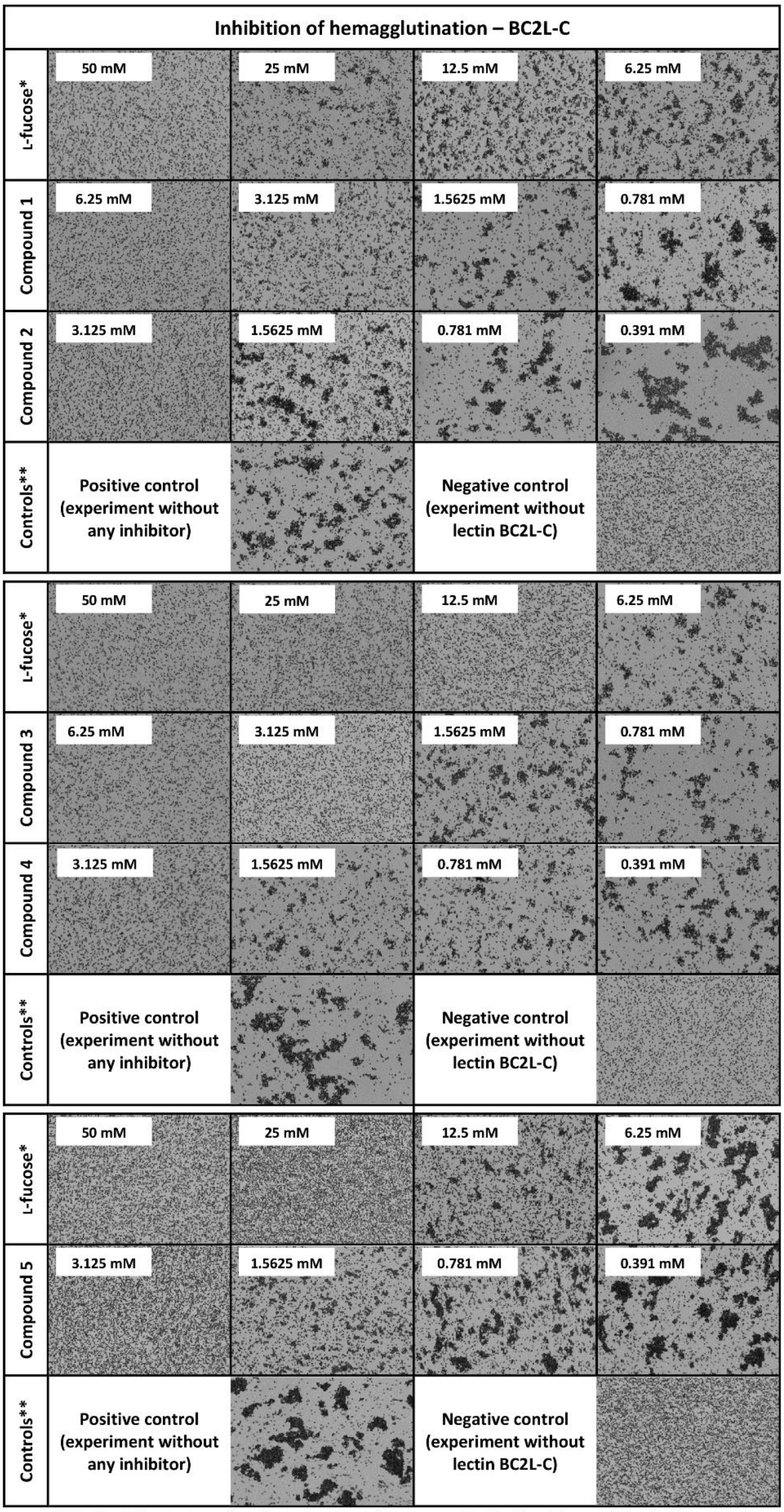

* Standard. Standard experiment was done anew for every used batch of protein or red blood cells.
** Controls were done anew for every used batch of protein or red blood cells.

S5. Influence of L-fucose, compounds **1**, **2**, **3**, **4** and **5** on hemagglutination caused by lectin BC2L-C.
